# Population vulnerability to COVID-19 in Europe: a burden of disease analysis

**DOI:** 10.1186/s13690-020-00433-y

**Published:** 2020-05-29

**Authors:** Grant M. A. Wyper, Ricardo Assunção, Sarah Cuschieri, Brecht Devleesschauwer, Eilidh Fletcher, Juanita A. Haagsma, Henk B. M. Hilderink, Jane Idavain, Tina Lesnik, Elena Von der Lippe, Marek Majdan, Milena S. Milicevic, Elena Pallari, José L. Peñalvo, Sara M. Pires, Dietrich Plaß, João V. Santos, Diane L. Stockton, Sofie Theresa Thomsen, Ian Grant

**Affiliations:** 1 Place and Wellbeing Directorate, Public Health Scotland, Glasgow, Scotland, UK; 2 Food and Nutrition Department, National Institute of Health Dr. Ricardo Jorge, Lisbon, Portugal; 3 Department of Anatomy, Faculty of Medicine and Surgery, University of Malta, Msida, Malta; 4 Department of Epidemiology and Public Health, Sciensano, Brussels, Belgium; 5 Department of Veterinary Public Health and Food Safety, Ghent University, Merelbeke, Belgium; 6 Data Driven Innovation Directorate, Public Health Scotland, Edinburgh, Scotland, UK; 7 Department of Public Health, Erasmus MC University Medical Center, Rotterdam, The Netherlands; 8 National Institute for Public Health and the Environment (RIVM), Bilthoven, The Netherlands; 9 National Institute for Health Development, Tallinn, Estonia; 10 National Institute of Public Health, Ljubljana, Slovenia; 11 Department of Epidemiology and Health Monitoring, Robert Koch Institute, Berlin, Germany; 12 Department of Public Health, Institute for Global Health and Epidemiology, Faculty of Health Sciences and Social Work, Trnava University, Trnava, Slovakia; 13 Faculty of Medicine University of Belgrade, Belgrade, Serbia; 14 MRC Clinical Trials and Methodology Unit, University College London, London, UK; 15 Unit of Noncommunicable Diseases, Department of Public Health, Institute of Tropical Medicine, Antwerp, Belgium; 16 National Food Institute, Technical University of Denmark, Lyngby, Denmark; 17 Exposure Assessment and Environmental Health Indicators, German Environment Agency, Berlin, Germany; 18 MEDCIDS, Department of Community Medicine, Information and Health Decision Sciences, Faculty of Medicine, University of Porto, Porto, Portugal; 19 CINTESIS, Centre for Health Technology and Services Research, Porto, Portugal; 20 Public Health Unit, ACES Grande Porto VIII - Espinho/Gaia, ARS Norte, Porto, Portugal

**Keywords:** COVID-19, Coronavirus, Burden of disease, DALY, YLD, Summary measures of population health, GBD, Vulnerability, European burden of disease network

## Abstract

**Background:**

Evidence has emerged showing that elderly people and those with pre-existing chronic health conditions may be at higher risk of developing severe health consequences from COVID-19. In Europe, this is of particular relevance with ageing populations living with non-communicable diseases, multi-morbidity and frailty. Published estimates of Years Lived with Disability (YLD) from the Global Burden of Disease (GBD) study help to characterise the extent of these effects. Our aim was to identify the countries across Europe that have populations at highest risk from COVID-19 by using estimates of population age structure and YLD for health conditions linked to severe illness from COVID-19.

**Methods:**

Population and YLD estimates from GBD 2017 were extracted for 45 countries in Europe. YLD was restricted to a list of specific health conditions associated with being at risk of developing severe consequences from COVID-19 based on guidance from the United Kingdom Government. This guidance also identified individuals aged 70 years and above as being at higher risk of developing severe health consequences. Study outcomes were defined as: (i) proportion of population aged 70 years and above; and (ii) rate of YLD for COVID-19 vulnerable health conditions across all ages. Bivariate groupings were established for each outcome and combined to establish overall population-level vulnerability.

**Results:**

Countries with the highest proportions of elderly residents were Italy, Greece, Germany, Portugal and Finland. When assessments of population-level YLD rates for COVID-19 vulnerable health conditions were made, the highest rates were observed for Bulgaria, Czechia, Croatia, Hungary and Bosnia and Herzegovina. A bivariate analysis indicated that the countries at high-risk across both measures of vulnerability were: Bulgaria; Portugal; Latvia; Lithuania; Greece; Germany; Estonia; and Sweden.

**Conclusion:**

Routine estimates of population structures and non-fatal burden of disease measures can be usefully combined to create composite indicators of vulnerability for rapid assessments, in this case to severe health consequences from COVID-19. Countries with available results for sub-national regions within their country, or national burden of disease studies that also use sub-national levels for burden quantifications, should consider using non-fatal burden of disease estimates to estimate geographical vulnerability to COVID-19.

## Background

In burden of disease studies, estimates of disability-adjusted life years (DALYs) are commonly used to assess the leading causes of burden amongst populations [1]. DALYs are composed of estimates of population health loss due to living with the consequences of morbidity and premature mortality. Years Lived with Disability (YLD) capture the morbidity (both the prevalence and severity of the disease) component of DALYs by estimating the number of years lost due to conditions diminishing the overall health status, and are a useful indicator to assess how impaired populations are due to living with the consequences of disease and injury [2].

Internationally, countries have reacted to the COVID-19 outbreak by introducing key public health non-pharmaceutical interventions (otherwise known as physical, or social, distancing) to protect vulnerable population groups [3]. Evidence has emerged to show that elderly people and those with pre-existing multi-morbid conditions may be at higher risk of developing severe health consequences from COVID-19 [4]. In Europe, 31% of the population are estimated to have a condition that is on the Government of the United Kingdom’s (UK) list of conditions at increased risk of severe health consequences from COVID-19 disease [5]. There is currently a disparity of comparable information across countries to objectively assess country-level vulnerability to COVID-19. However, there is a wealth of data on population structure, health status and causes of health loss in countries, which can be obtained from the Global Burden of Disease (GBD) study [6]. These data can be used to approximate how vulnerable populations are, particularly by focusing on the population share of elderly residents and the YLD for health conditions that have been identified as potentially linked to severe illness from COVID-19. This is of particular relevance for European countries, as increases in lifespan have resulted in increasingly ageing populations living with effects of non-communicable diseases, multi-morbidity and frailty [7].

The aim of this study was to identify the countries across Europe that have populations at highest risk for severe disease progression after COVID-19 infection by using estimates of population structure and YLD for health conditions linked to severe illness from COVID-19. This study was carried out using data from GBD 2017 for the reference year 2017, considering two measures of vulnerability: (i) rate of elderly population; and (ii) rate of YLD for health conditions identified at risk of severe health consequences from COVID-19.

## Methods

### Data

The GBD Results Tool [8] was used to extract Years Lived with Disability (YLD) estimates for both sexes, age-groups (all ages; 70 years and above; and 80 years and above) and GBD 2017 level 3 cause [9] for each country defined as residing in Central, Eastern and Western Europe (*N* = 45 countries). Estimates were considered for the constituent nations of the United Kingdom (UK): England; Northern Ireland; Scotland; and Wales, rather than the UK as a whole. In this study, hereafter, the elderly population denotes the age-group 70 years and above.

Data were retained for specific causes based on guidance from the UK Government (as at 30th March 2020) on those health conditions that indicated a risk of severe health consequences from COVID-19 [10]. Two groups were defined: individuals aged 70 years and above, and those under 70 years that have one or more pre-existing underlying health condition. The guidance provided by the UK Government is outlined in the Supplementary Appendix and the list of pre-existing conditions were mapped to the GBD 2017 cause list (Table 1).

**Table 1:** Mapping of UK Government guidance on pre-existing medical conditions at risk of severe illness from COVID-19 to the GBD 2017 cause list

Pre-existing health condition(s)	GBD mapped cause(s)
All health conditions	All-causes
Chronic respiratory diseases	Chronic obstructive pulmonary disease
	Pneumoconiosis
	Asthma
	Interstitial lung disease and pulmonary sarcoidosis
	Other chronic respiratory diseases
Chronic heart disease	Cardiovascular diseases (chronic and acute)
Chronic kidney disease	Chronic kidney disease
Chronic liver disease	Cirrhosis and other chronic liver diseases
Chronic neurological conditions	Alzheimer’s disease and other dementias
	Parkinson’s disease
	Epilepsy
	Multiple sclerosis
	Motor neuron disease
	Other neurological disorders
Diabetes	Diabetes mellitus
Problems with spleen	Sickle cell disorders
	Sickle cell trait
Cancer undergoing active chemotherapy or radiotherapy	All cancer types
Cancers of the blood or bone marrow	
Seriously overweight	Covered in mapping for chronic respiratory diseases, cardiovascular diseases, chronic kidney disease, cirrhosis and chronic liver diseases, and diabetes
Severe chest conditions such as cystic fibrosis or severe asthma	Covered in mapping for chronic respiratory diseases
Organ transplant and remain on ongoing immunosuppression medication	Covered in mapping for chronic kidney disease
Severe disease of body systems	Covered in mapping from all pre-existing health conditions
Those who are pregnant	Not covered

Some emerging evidence has considered obesity and hypertension as independent risk factors for severe health consequences from COVID-19 [11, 12]. However, we do not consider them separately in this study as the vast majority of disease outcomes associated with these risk factors are included in the mapping to the GBD cause list (Table 1). For hypertension, all disease outcomes linked to the high systolic blood pressure risk factor are included (cardiovascular diseases and chronic kidney disease in Table 1). In addition, GBD include separate estimates for hypertensive disease and these are included within cardiovascular diseases. The disease outcomes associated with obesity are also all covered in the mapping to the GBD cause list with the exception of Gout. Gout accounted for only 0.2% (95% uncertainty interval: 0.15–0.25%) of total YLD in GBD European Region in 2017 [8].

A permalink to the GBD Results Tool [8] query that were used to generate the data used in this study are outlined in the Data Availability section. Additionally, data on the total 2017 resident populations and population aged 70 years and above for each country were sourced from the Global Health Data Exchange (GHDx) [13]. These population denominators were used in the production of GBD 2017 estimates.

### Analyses

Descriptive summaries were calculated for the proportion of elderly population, and YLD for COVID-19 vulnerable health conditions were described using crude rates per 100,000 population. The numerators for the population proportion calculations were based on elderly populations, whereas the YLD rate calculation numerators were based on population totals. Denominators were based on the all ages population data sourced from GHDx [13].

Each measure was divided into tertiles (three binned categories: low; mid; and high). These categories were calculated to determine three equal size groups of vulnerability. Bivariate groupings were established by considering the overlapping of the measures and were depicted in a scatter plot to identify groups of countries, both in terms of the proportion of elderly population and the rate of YLD for conditions associated with worse COVID-19 prognosis. Spearman’s rank correlation coefficient (ρ) was used to describe the correlation between the percentage of elderly population and the rate of YLD for COVID-19 vulnerable health conditions.

## Results

### Proportions of elderly population by country

The five countries with the highest proportions of elderly residents (aged 70 years and above) were: Italy (16.4%); Greece (16.2%); Germany (15.4%); Portugal (15.3%); and Finland (14.7%) (Table 2). Conversely, the countries with the lowest proportions of elderly population were Israel (7.7%); Moldova (8.2%); Russian Federation (8.8%); Macedonia (8.8%); and Albania (9.1%). The ratio of the country with the highest (Italy) and lowest (Israel) proportion of elderly residents was 2.14, indicating over a two-fold difference between the countries.

**Table 2:**
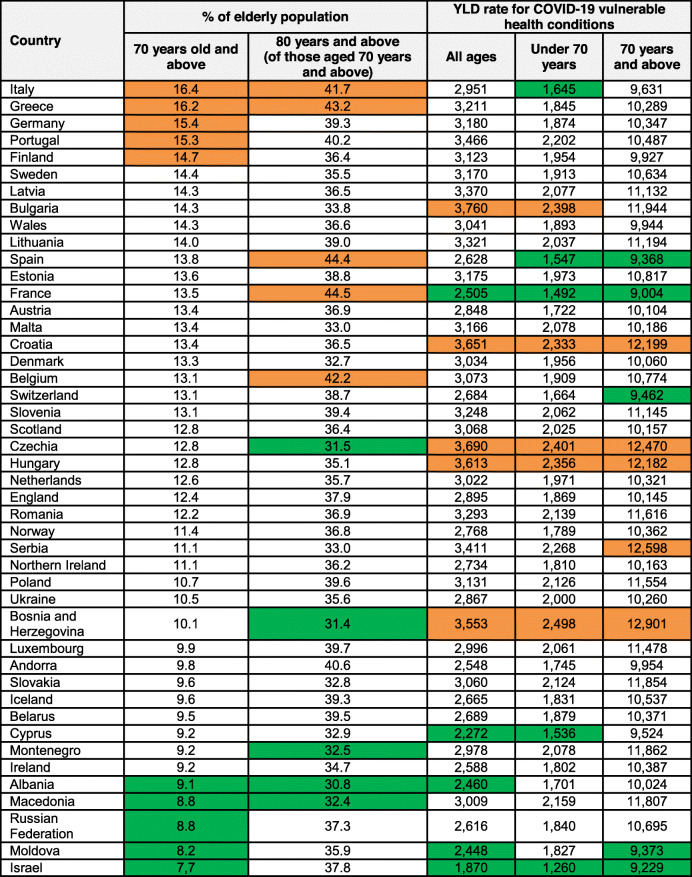
Summary of percentage of elderly population and YLD rates for COVID-19 vulnerable health conditions, by country, 2017

Country order is based on the descending proportion of elderly residents. Cells shaded in orange represent the highest values within each metric, with cells shaded in green representing the lowest value

When looking at stratified differences within the elderly age-group, the five countries with the highest percentage of population aged 80 years and above were: France (44.5%); Spain (44.4%); Greece (43.2%); Belgium (42.2%) and Italy (41.7%). The five countries with the lowest percentage of population aged 80 years and above were: Albania (30.8%); Bosnia and Herzegovina (31.4%); Czechia (31.5%); Macedonia (32.4%); and Montenegro (32.5%). Between the country with the highest (France) percentage of population aged 80 years and above and lowest (Albania), there was an absolute difference of 13.7%.

### Rate of YLD for COVID-19 vulnerable health conditions

When the rate of YLD for health conditions associated with higher COVID-19 vulnerability was assessed for all ages, the five countries with the highest rates per 100,000 population were: Bulgaria (3760); Czechia (3690); Croatia (3651), Hungary (3613); and Bosnia and Herzegovina (3553) (Table 2). The five countries with the lowest rates were: Israel (1870); Cyprus (2272); Moldova (2448); Albania (2460); and France (2505). There was a rate ratio of 2.01 between the country with the highest rate (Bulgaria) and the country with the lowest rate (Israel).

Insights into rates of YLD for health conditions indicating higher COVID-19 vulnerability for those under 70 years and elderly residents were that there were four countries that were common amongst the leading five countries in both age-groups. These countries were: Czechia, Croatia, Hungary and Bosnia and Herzegovina. Of the five countries with the lowest rates in the under 70 years and elderly age-groups, there were three countries that were common: Israel, France and Spain.

### Summary of combined vulnerability

**Fig. 1:**
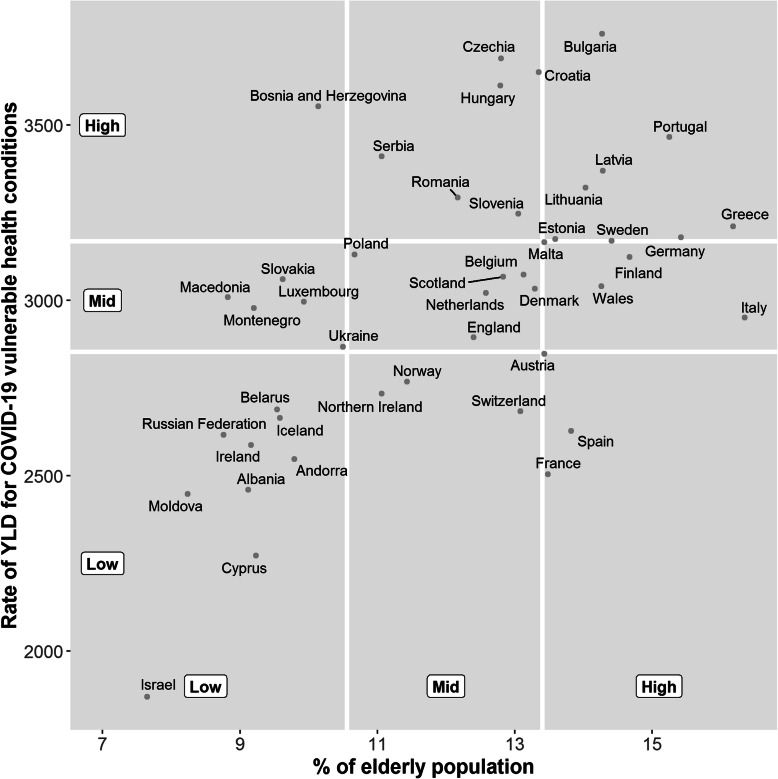
Scatter plot of percentage of elderly population versus rate of YLD for COVID-19 vulnerable health conditions for European countries. Rates described are crude rates per 100,000 population. White vertical and horizontal gridlines indicate the tertile dividing lines for the measures: percentage of elderly population; and rate of YLD for COVID-19 vulnerable health conditions, respectively

There was a moderate association (ρ = 0.54) between the percentage of elderly population and the rate of YLD for COVID-19 vulnerable health conditions. A bivariate analysis indicated that the countries which had high proportions of elderly population and high rates of YLD for COVID-19 vulnerable health conditions were: Bulgaria; Portugal; Latvia; Lithuania; Greece; Germany; Estonia; and Sweden. Conversely, the countries with the lowest proportions of elderly population and lowest rates of YLD for COVID-19 vulnerable health conditions were: Israel; Cyprus; Moldova; Albania; Andorra; Ireland; Russian Federation; Iceland; and Belarus. Bosnia and Herzegovina had a high rate of YLD for COVID-19 vulnerable health conditions, but a relatively low proportion of elderly population. On the other hand, Spain, France and Austria all had high proportions of elderly population but a relatively low rate of YLD for COVID-19 vulnerable health conditions (Fig. 1).

## Discussion

### Summary of findings

This study set out to establish which countries across Europe had populations that were most likely to be vulnerable to severe health consequences as a result of COVID-19 infection. This assessment was made using data on population age structure, and data on YLD for health conditions identified as increasing the risk of COVID-19 severity, the latter illustrating the extent to which populations are vulnerable through living with ill-health due to causes of disease.

Estimates of vulnerability to COVID-19 using elderly population share indicated that the countries with the highest proportions of elderly residents were Italy, Greece, Germany, Portugal and Finland. When assessments of population-level YLD rates for COVID-19 vulnerable health conditions were made the highest rates were observed for Bulgaria, Czechia, Croatia, Hungary and Bosnia and Herzegovina. Our bivariate analysis indicated that the countries which had high rates across both measures of vulnerability were: Bulgaria; Portugal; Latvia; Lithuania; Greece; Germany; Estonia; and Sweden.

Whilst these findings indicate population-level vulnerability due to health loss suffered, they do not take into account other important factors such as country and sub-national responses to the COVID-19 outbreak through public health non-pharmaceutical interventions. Neither do they take into account factors such as: population density, the capacity or ease of access to health and social care services and the disruption to existing services due to the COVID-19 crisis, all of which will have a significant impact on the extent to which vulnerable populations are adequately protected from harm. This may partly explain why countries identified in this analysis with high and low vulnerability to severe health consequences from COVID-19 do not always correspond with those countries in Europe with the highest and lowest case fatality ratios due to COVID-19 [14]. For example, within the Baltic states Latvia and Estonia have high vulnerability as measured on both indicators. However, Latvia responded to the crisis quickly by closing their borders and implementing restrictive measures much faster than Estonia, and case fatality rates are higher in Estonia [14–16]. This example highlights that a number of additional factors could contribute to differences between vulnerability and extent of adverse consequences, including: care identification and under-reporting, the speed at which countries introduced restrictive measures, and restrictions on air travel. The use of summary health indicator such as YLD to identify severe health consequences from COVID-19 infections should be regarded as just one of the elements that need to be taken into account in a complete risk assessment of vulnerability.

### Strengths and limitations

The study was carried out using estimates from GBD 2017, which is a widely used and well-established mechanism that has methodological consistency when producing estimates for individual countries [6]. The use of GBD 2017 is advantageous as estimates are publically accessible, which allows for the rapid assessments of impact in response to public health emergency scenarios, such as the COVID-19 outbreak. Our findings are comparable on a like-for-like basis across countries. However, data sources that are fed into the modelling process for country-level estimates can vary based on location, therefore there is a risk that some of the differences which we observe may be attributed to the use, or omission, of high quality data sources [17]. We have opted not to include estimates of uncertainty in our estimates. Uncertainty intervals in the GBD study can often be wide, representing large degrees of uncertainty, so users of these results must bear in mind that these findings relate to the best available point-estimate. To retain consistency with estimates of YLD from GBD 2017, data on population size and structure was obtained from GHDx [13] which may differ from nationally produced estimates.

Previous research has suggested that the assumption of fixed severity distributions across countries may be unreasonable [18]. In our study of COVID-19 related vulnerable conditions, we did not include some of the leading causes of YLD, such as major depressive disorders and substance use disorders, which are thought to be the most likely to be affected by this assumption. Thus, our COVID-19 vulnerable conditions analysis may be less affected by this assumption [19]. Additionally, our study has assumed that the extent of vulnerability to COVID-19 can be determined by disability weights. For example, on average a greater weight would be given to those suffering from chronic obstructive pulmonary disease than to ischaemic heart disease [20]. This assumption may be problematic if the risk of COVID-19 associated with each health condition is not representative of relative differences in disability weight between causes. Also, particular combinations of disease may result in higher risks of consequences of COVID-19, while all combinations are in this approach assumed to have a similar effect.

We have used YLD as a proxy for the severity of the selected vulnerable health conditions as YLD includes a weighting of the severity of diseases stages i.e. a weighted prevalence. We have chosen to explore the aim of the YLD summary measure to combine all conditions, rather than examine the impact of individual causes. We acknowledge that using disease prevalence data from GHDx could add further insight into quantifying the disease specific implications of severe health consequences from COVID-19. However, since prevalence gives equal weighting to each condition, we did not consider prevalence as useful for summary analyses as YLD which allows a weighted sum of prevalence of different diseases. Further analysis has previously been carried out elsewhere to explore using prevalence to quantify the risk for severe health consequences from COVID-19 infection to enhance assessment of a health systems vulnerability to COVID-19 [5].

### Implications for policy and research

Our findings have important implications for decision-making and for future research. In our assessment of vulnerability, we have highlighted the countries in Europe with populations that are elderly, and vulnerable as a result of reduced health due to certain health conditions. From a decision-making perspective, this effectively communicates how locations can be assessed on a relative scale of risk of severe illness due to COVID-19. Although, at time of this publication, many countries have now employed public health non-pharmaceutical interventions for over two months, there are still many uncertainties about the further evolution of the pandemic and the virus itself. Therefore, until an effective vaccination or treatment is available, our findings highlight which countries have populations that are at highest risk and therefore should be prioritising the shielding of vulnerable individuals. Doing so can alleviate the extent to which essential healthcare services are overwhelmed, which will also contribute to curtailing the indirect impact of COVID-19. Still, our results should not be used as rationale for countries to justify a relaxing of existing non-pharmaceutical interventions. The countries we identified as having indicators of low vulnerability should not be complacent, as doing so would have severe adverse consequences. Our findings also have important implications if there are positive breakthroughs in the development of a vaccine that is both safe and effective, as they highlight which countries may benefit from it the most.

As research begins to focus on the evaluation of the impact of public health non-pharmaceutical interventions, an important aspect will be to establish baseline measures of risk to severe illness of COVID-19. Our findings provide an opportunity for this, particularly when assessing the factors for success of these interventions in populations that were facing similar levels of population vulnerability.

## Conclusion

Our findings have highlighted that routine data on population structure can be usefully extended by using estimates of YLD to consider how populations are impaired by living with the consequences of ill-health due to causes of disease and injury. Countries with available estimates for sub-national regions within their country, or national burden of disease studies that also estimate at sub-national levels should consider using non-fatal burden of disease estimates to estimate geographical vulnerability to COVID-19.

## Supplementary information

**Additional file 1.** Guidance on social distancing from the United Kingdom Government.

## Data Availability

The datasets used in this research study are all publically available. The permalink to data query used to obtain estimates of YLD is: http://ghdx.healthdata.org/gbd-results-tool?params=gbd-api-2017-permalink/376d9a9ad8401f49f104650fab0b9305.

## Abbreviations

COVID-19: Coronavirus Disease 2019
DALYs: Disability-Adjusted Life Years
GBD: Global Burden of Disease
GHDx: Global Health Data Exchange
UK: United Kingdom
YLD: Years Lived with Disability
