# Correction to: Population vulnerability to COVID-19 in Europe: a burden of disease analysis

**DOI:** 10.1186/s13690-020-00437-8

**Published:** 2020-06-18

**Authors:** Grant M. A. Wyper, Ricardo Assunção, Sarah Cuschieri, Brecht Devleesschauwer, Eilidh Fletcher, Juanita A. Haagsma, Henk B. M. Hilderink, Jane Idavain, Tina Lesnik, Elena Von der Lippe, Marek Majdan, Milena S. Milicevic, Elena Pallari, José L. Peñalvo, Sara M. Pires, Dietrich Plaß, João V. Santos, Diane L. Stockton, Sofie Theresa Thomsen, Ian Grant

**Affiliations:** 1 Place and Wellbeing Directorate, Public Health Scotland, Glasgow, Scotland, UK; 2 Food and Nutrition Department, National Institute of Health Dr. Ricardo Jorge, Lisbon, Portugal; 3 Department of Anatomy, Faculty of Medicine and Surgery, University of Malta, ida, Malta; 4 Department of Epidemiology and Public Health, Sciensano, Brussels, Belgium; 5 Department of Veterinary Public Health and Food Safety, Ghent University, Merelbeke, Belgium; 6 Data Driven Innovation Directorate, Public Health Scotland, Edinburgh, Scotland, UK; 7 Department of Public Health, Erasmus MC University Medical Center, Rotterdam, The Netherlands; 8 National Institute for Public Health and the Environment (RIVM), Bilthoven, The Netherlands; 9 National Institute for Health Development, Tallinn, Estonia; 10 National Institute of Public Health, Ljubljana, Slovenia; 11 Department of Epidemiology and Health Monitoring, Robert Koch Institute, Berlin, Germany; 12 Department of Public Health, Institute for Global Health and Epidemiology, Faculty of Health Sciences and Social Work, Trnava University, Trnava, Slovakia; 13 Faculty of Medicine University of Belgrade, Belgrade, Serbia; 14 MRC Clinical Trials and Methodology Unit, University College London, London, UK; 15 Unit of Noncommunicable Diseases, Department of Public Health, Institute of Tropical Medicine, Antwerp, Belgium; 16 National Food Institute, Technical University of Denmark, Lyngby, Denmark; 17 Exposure Assessment and Environmental Health Indicators, German Environment Agency, Berlin, Germany; 18 MEDCIDS, Department of Community Medicine, Information and Health Decision Sciences, Faculty of Medicine, University of Porto, Porto, Portugal; 19 CINTESIS, Centre for Health Technology and Services Research, Porto, Portugal; 20 Public Health Unit, ACES Grande Porto VIII - Espinho/Gaia, ARS Norte, Porto, Portugal

**Correction to: Archives of Public Health (2020) 78:47**

**https://doi.org/10.1186/s13690-020-00433-y**

Following publication of the original article [1], the authors identified an error in the author name of Brecht Devleesschauwer.

The incorrect author name is: Brecht Devleeschauwer

The correct author name is: Brecht Devleesschauwer

The author group has been updated above and the original article [1] has been corrected.
