# Supplementary material for: Population vulnerability to COVID-19 in Europe: a burden of disease analysis
Source: Arch Public Health. 2020 May 29;78:47. doi: 10.1186/s13690-020-00433-y (PMC7256342; doi:10.1186/s13690-020-00433-y)
Supplement: Supplementary file 1 — Additional file 1. Guidance on social distancing from the United Kingdom Government. [file 13690_2020_433_MOESM1_ESM.docx]

**Supplementary Material**

**Guidance on social distancing for everyone in the United Kingdom**

| Source: Public Health England. Guidance on social distancing for everyone in the UK. United Kingdom Government. <https://www.gov.uk/government/publications/covid-19-guidance-on-social-distancing-and-for-vulnerable-people/guidance-on-social-distancing-for-everyone-in-the-uk-and-protecting-older-people-and-vulnerable-adults>. Accessed 10 Apr 2020.  We are advising those who are at increased risk of severe illness from coronavirus (COVID-19) to be particularly stringent in following social distancing measures.  This group includes those who are:   - aged 70 and above (regardless of medical conditions) - under 70 with an underlying health condition listed below (i.e. anyone instructed to get a flu jab as an adult each year on medical grounds): - chronic (long-term) respiratory diseases, such as asthma, chronic obstructive pulmonary disease (COPD), emphysema or bronchitis - chronic heart disease, such as heart failure - chronic kidney disease - chronic liver disease, such as hepatitis - chronic neurological conditions, such as Parkinson’s disease, motor neurone disease, multiple sclerosis (MS), a learning disability or cerebral palsy - diabetes - problems with your spleen – for example, sickle cell disease or if you have had your spleen removed - a weakened immune system as the result of conditions such as HIV and AIDS, or medicines such as steroid tablets or chemotherapy - being seriously overweight (a body mass index (BMI) of 40 or above) - those who are pregnant   Note: there are some clinical conditions which put people at even higher risk of severe illness from COVID-19. If you are in this category, next week the NHS in England will directly contact you with advice about the more stringent measures you should take in order to keep yourself and others safe. For now, you should rigorously follow the social distancing advice in full, outlined below.  People falling into this group are those who may be at particular risk due to complex health problems such as:   - people who have received an organ transplant and remain on ongoing immunosuppression medication - people with cancer who are undergoing active chemotherapy or radiotherapy - people with cancers of the blood or bone marrow such as leukaemia who are at any stage of treatment - people with severe chest conditions such as cystic fibrosis or severe asthma (requiring hospital admissions or courses of steroid tablets) - people with severe diseases of body systems, such as severe kidney disease (dialysis) |
| --- |
